# Regulatory review and governance gaps for genetically engineered microbes

**DOI:** 10.3389/fgene.2026.2008399

**Published:** 2026-10-28

**Authors:** Jennifer Kuzma, Christopher Cummings, Khara Grieger

**Affiliations:** 1 School of Public and International Affairs, North Carolina State University, Raleigh, NC, United States; 2 Genetic Engineering and Society (GES) Center, North Carolina State University, Raleigh, NC, United States; 3 Department of Applied Ecology, North Carolina State University, Raleigh, NC, United States; 4 North Carolina Plant Science Initiative, North Carolina State University, Raleigh, NC, United States

**Keywords:** biotechnology, genetically engineered microbes, governance, policy recommendations, regulation

## Abstract

Living genetically engineered microorganisms (GEMs) are increasingly used in consumer products and environmental applications, yet U.S. biotechnology regulation relies on statutes that predate their development or are designed for other types of products. This article examines oversight of living GEMs through 3 case studies of consumer and environmental GEM applications. Across these cases, federal premarket review was very limited or absent because oversight depended on distinctions such as the type of modification, the developer’s product classification, or marketing claims. These regulatory pathways may not always align uniformly with the potential health and environmental risk profiles. While not all GEMs require the same level of oversight, current regulatory gaps have allowed for the large-scale, open release of these GEMs without substantial federal oversight. The article recommends baseline federal notification, changes to statutes or regulations to strengthen regulatory foundations, greater transparency, independent evaluation, and post-market monitoring, while also preserving efficient pathways for lower-risk applications. These enhancements to the regulation of GEMs will become increasingly important for effective risk governance as consumer and environmental products incorporate advanced synthetic biology approaches.

## Introduction

Living, genetically engineered microbes (GEMs)^1^ are now appearing in daily consumer products and large-scale environmental applications, after decades mainly of containment in laboratory production settings or small-scale field trails. As GEMs are used more widely and in open systems, it is important to ensure their safety, sustainability, and responsible use. Potential environmental and health risks that need to be considered for open release of living GEMs include, for example, horizontal gene transfer or foreign DNA uptake that may lead to adverse consequences for other microbial or non-microbial species; consumption of the GEM by non-target organisms, wildlife, and/or humans; and other exposure pathways that may impact human, animal, environmental, or microbiome health. Microbial ecology and dynamics within animal and environmental ecosystems will play a role in the mitigation or magnification of such risks, and an understanding of GEM mechanisms of survival and genetic exchange within the environment will be important for assessing them. It is important to understand a number of complex processes that extend across and in between scales, such as host dietary and societal factors that can shape microbiome composition and evolution (see de Sousa et al. (2023) for more details, as well as Tokuda and Shintani (2024) for details on mechanisms underlying horizontal gene transfer and the role of mobile genetic elements in evolution and adaptation).

In addition, advances in synthetic biology have enabled the construction of GEMs containing genes made from synthetic DNA (DNA sequences and genes not found in nature) and foreign biochemicals such as amino acids and DNA base pairs not found in nature (xenobiology) (NASEM, 2026). As discussed further below, these developments expose the limitations of regulatory approaches that rely on fixed categorical definitions or the source of genetic material (NASEM, 2026).

For 40 years, GEMs have been regulated under the Coordinated Framework for the Regulation of Biotechnology (CFRB), which divided oversight among three main federal departments or agencies based on the product’s intended use (White House, 1986). The CFRB is a policy document that instructed the federal agencies in 1986 to use existing laws for chemicals, drugs, and plant pests to cover the products of biotechnology. No new laws for biotechnology oversight were passed in the United States or have been since, and most of the statutes apply to static, chemical products not living organisms. As a result, some emerging GEM products do not fit into the CFRB scheme, even with its evolution and updates (Kuzma, 2023). This article covers three products that have entered the open market without pre-market federal regulatory review, including one used as a “food ingredient”, one as a “cosmetic”, and one for agricultural release. After a brief introduction to GEMs regulation under the CFRB, these products are discussed to illustrate gaps in oversight under the CFRB and discuss proposals for strengthening regulatory foundations. Addressing these shortcomings in oversight will be even more important as GEMs are made with increasingly complex synthetic biology methods and deployed more widely in open environmental systems or consumer products. Each case study thus ends by considering how these gaps may present additional challenges as emerging synthetic biology approaches are applied to the development of GEMs.

## Regulation of GEMS

### Environmental protection agency (EPA)

The Environmental Protection Agency (EPA) regulates “intergeneric” (engineered to contain foreign genes from different genus) microorganisms under the Toxic Substances Control Act (TSCA). Manufacturers must submit a Microbial Commercial Activity Notice (MCAN) before commercialization. For environmental release of GEMs, a permit is required under a Toxics Environmental Release Application (TERA). The EPA reviews environmental persistence, gene transfer potential, and ecological toxicity. Intergeneric GEMs regulated under TSCA have been released in small-scale field trials since the mid-1980s (Wozniack et al., 2012) although widespread use did not take off until recently. Previous applications include bioremediation, nitrogen fixation, and pest-control. Notably, GEMs that are not intergeneric, but rather “intrageneric” (engineered with genes from same genera) are not regulated under TSCA. This is elaborated further in subsequent sections.

The EPA also regulates microbes under the Federal Fungicide, Insecticide, and Rodenticide Act (FIFRA) regulations if they are claimed to kill pests. GE microbial pest-control agents that have been reviewed and registered by the EPA include GE *Bacillus thuringiensis* (Bt) to control agricultural pests like corn rootworm and GE *Agrobacterium radiobacter* to prevent crown gall disease on trees and plants (Wozniack et al., 2012). EPA FIFRA review and registration requires pre-market data for human health hazards, non-target organism toxicity, and environmental fate.

### Food and drug administration (FDA)

The FDA oversees GEMs used in food additives, dietary supplements, human drugs, and cosmetics under the Federal Food Drug and Cosmetic Act (FDCA). FDA approval processes for drugs and biologics are intensive, requiring staged *in vitro,* animal, and clinical testing that can take years to decades. For example, a live oral vaccine for cholera, Vaxchora, is derived from using recombinant DNA technology to remove the toxin gene from *Vibrio chlorae* and add a mercury resistance marker gene. This product has been approved by the FDA as a biologic after extensive animal and clinical trials (FDA-DHHS, 2016; 2024; McCarty et al., 2022). This is an example of a live GEM being used in medicine through oral delivery. FDA also regulates products derived and purified from GEMs in the laboratory (e.g., antibiotics, biologics, drugs) to ensure safety, quality, and efficacy. Other live GEMs are being developed and tested in clinical trials for disease treatment (Gulig et al., 2024; Santos-Beneit, 2024). The key to FDA regulatory coverage for drugs and biologics depends on the claims to prevent or treat diseases or medical conditions. We expand on this point in subsequent sections.

FDA regulates food additives under the FDCA as well. If a substance added to food is new and not generally regarded as safe (GRAS), it is reviewed by FDA prior to market entry through a food additive petition under the FDCA (Snitkoff and David, 2026). Food additive petitions contain data on identity and composition, exposure estimates, safety at expected exposures, and proposed tolerance levels, as well as environmental assessments (21 CFR Part 171). FDA makes a determination whether or not the product is safe for its intended use. There are many products derived from GEMs in the laboratory that have been regulated as food additives, including sweeteners, flavorings, enzymes, colors, and vitamins. Live GEMs have not currently been approved by FDA as food additives to our knowledge but have entered the market under other classifications. For example, food ingredients can by-pass the rigorous food-additive regulatory pathway by being categorized as GRAS which is either determined by “scientific consensus” or common use already in food. GRAS determination can take place either through developer self-determination or through notification to FDA.

For cosmetics and dietary supplements, the FDA lacks pre-market approval authority but instead relies on developers doing their own safety analysis and post-market recall authority under the FDCA or Dietary Supplements Health and Education Act (DSHEA). Live GEMs have entered consumer products under “cosmetics,” as expanded upon further below.

### Department of agriculture (USDA)

The USDA Animal and Plant Health Inspection Service (APHIS) regulates GEMs that are considered plant pests under the Plant Protection Act (PPA) (Wozniack et al., 2012). The USDA primarily regulates GEMs by issuing movement, importation, and environmental release permits. For example, a Citrus Tristeza Virus (CTV) GE strain, genetically modified with defensin proteins from spinach plants and designed to kill Citrus Greening Disease caused by a bacteria, was regulated by the USDA under the PPA. CTV is itself a plant pathogen, and therefore the genetically engineered CTV came under USDA’s authority under the PPA. It also was regulated under EPA’s authority under FIFRA given its intended use to kill plant pests. The genetically modified CTV underwent review via a USDA plant pest risk assessment mandated under the PPA, in order to receive an environmental release permit. The GEM also was reviewed under National Environmental Protection Act via an extensive Environmental Impact Statement. However, USDA’s regulatory authority focuses on risks to plants via pests, and some non-target organism risks (e.g., risks to insects and other organisms consuming such plants or to microbiomes) were not assessed (Eckerstorfer et al., 2024; Dolezel et al., 2024). The authorized permit includes limited, but large-scale releases in citrus-growing regions in FL, up to 513,500 acres in all 67 counties of the state (USDA, 2020).

## Results: cases of regulatory loopholes

The above review of GEM regulations illustrates that many GEM products are well-covered by the U.S. CFRB. If a GEM is clearly marketed as a pesticide or drug/biologic, or has plant pest properties, it will undergo pre-market review, including the need for risk or safety analyses that illustrate to the federal agencies that the health and environmental risks are reviewed, minimal, and not unreasonable. However, there are significant gaps in the CFRB in large part due to its reliance on older product-based statutes and regulations. Three recent cases of GEMs that are discussed below are used in open-release or consumer settings and highlight these deficiencies. The selection of the cases was based on their relevance to current, real-world applications (i.e., recent cases involving products already on the market) and to illustrate gaps in the regulatory system for GEM products under wide-scale consumer or environmental use. Further, we chose cases that involve the potential for open release or consumer use, rather than applications limited to closed-containment settings. Cases were identified through searches of peer-reviewed literature, public websites, and other publicly accessible online sources.

In two cases, product-advertising approaches allowed companies to bypass pre-market regulatory review pathways, while in another case, new gene-editing technologies enabled the creation of a GEM that slips through a regulatory crack. Although the regulatory system has been purported to be “science-based,” an argument can be made that the lack of sufficient oversight in these cases is not science-based, as risk is not determined by claims or the source of genetic material (e.g., one could increase a toxin or an allergen in a plant by a cisgenic, small mutation) (NRC, 2000).

In the following sections, each case is described followed by an analysis of key regulatory gaps and broader implications for the field of GEMs. Although these three cases do not represent all GEMs in development or on the market, they were selected to provide a examination of currently marketed living GEM products across different product categories, enabling the authors to draw broader insights beyond the individual case studies for these types of products.

### GE nitrogen-fixing bacteria

Nitrogen fixation is essential for crop growth, and although some plants can fix atmospheric nitrogen on their own, important crops like corn cannot and rely on the application of chemical fertilizers which can have detrimental environmental impacts. Pivot Bio developed a product called PROVEN® 40 that includes genetically engineered, plant root-associated bacteria that helps crops like maize fix nitrogen (Wen et al., 2021; Ahmad et al., 2024). Two bacteria *Kosakonia sacchari* and *Klebsiella variicola*, were isolated from maize roots and mapped out to target genes for nitrogenase production, nitrogen fixation, ammonium excretion, and root colonization. CRISPR gene-editing and other biotechnology techniques were used to optimize the bacteria for nitrogen fixation, free ammonium production and root colonization. Genes for downregulation of nitrogen fixation in the wild type bacteria were disabled using gene editing, so that the GEMs could constitutively fix nitrogen for the crop throughout its lifecycle. There are significant environmental benefits associated with these GEMs, as they are estimated to replace over 30 pounds per acre of synthetic, chemical fertilizer when applied to seed (out of about 200 lbs/acre fertilizer typically applied) (Pivot Bio, 2026). After launching PROVEN 40 in 2019, by 2023 it had been used on 5 million acres in the U.S. (Miller, 2023).

The GE bacterial strains used in PROVEN do not contain sequences from other species, but rather endogenous genes were edited to make the product. Therefore, it is an intrageneric GEM and falls outside of EPA’s TSCA regulations (as discussed above). The product did not require a MCAN, TERA, or any premarket review by the EPA. The product is also not a pesticide or a plant pest, so it was not covered by EPA’s FIFRA regulations or USDA’s PPA permitting process. EPA FIFRA regulations exclude “A plant inoculant product consisting of microorganisms to be applied to the plant or soil for the purpose of enhancing the availability or uptake of plant nutrients through the root system” (40 CFR part 152.6). Thus, PROVEN 40 required no mandatory, federal regulatory safety review prior to marketing (Ahmad et al., 2024; Brewer et al., 2026). The company states that FDA considered the product under a voluntary consultation process to investigate whether the food/feed portions of crops have detectable levels of microbes (Jensen, 2025), although documentation or studies from this consultation could not be found by the authors online. PROVEN 40 is classified differently by state agencies as a soil amendment, fertilizer, plant amendment, or soil conditioner; and state requirements for registration, efficacy data, labeling, product renewals, and tonnage reports vary (Jensen, 2025).

In the absence of required regulatory safety or risk assessments, the author found brief descriptions of safety studies conducted on PROVEN in the peer reviewed literature. Wen et al. (2021), a paper authored by Pivot Bio personnel, include a section on biosafety studies, in which the authors state *“As certain K. variicola strains have been isolated in healthcare environments as opportunistic pathogens, we thoroughly investigated the toxicity and pathogenicity of Kv137-1036 in a mammalian system” (p 3270*). *Klebsiella variicola* is a human pathogen of increasing medical concern, showing antibiotic resistance and causing respiratory, wound, urinary tract, and other infections in hospital settings (Potter et al., 2018; Barrios-Camacho et al., 2019). Outside of hospital-acquired infections, *K. variicola* exposure could also occur via waterways, food, livestock, and wildlife (Barrios-Camacho et al., 2019). While Wen et al. (2021) state the thoroughness of their toxicity and pathogenicity studies, they list six studies conducted on rats or rabbits, five of which were short duration irritation (skin and eye) or toxicity studies (oral route) and only one focused on pathogenicity. The pathogenicity study looked at one route of exposure, inhalation, in which *“17 rats of each sex) received a pulmonary dose of 0.15 mL containing 1.0 × 10* ^*1*^ *viable cells of the test microorganism Kv137-1036”* (Wen et al. 2021, p 3274). After pulmonary inoculation, the authors state in the methods that:

*“Samples of lung, blood, brain, kidney, liver, lymph nodes, spleen, and cecum contents were collected from all animals at their scheduled sacrifice at days 1, 3, 7, 15, and 22/23. Body weights were recorded from surviving animals on days 1, 3, 8, 15, and prior to sacrifice (day 22/23). At each sacrifice, blood and organ tissues were plated to determine the amount of Kv137-1036 present.”*

Results are discussed in two places in the article, although the detailed data are not shown. In a supplemental table to the article, results are described as:

*“No mortalities nor clinical observations attributed to pulmonary administration of Kv137-1036 were observed. Mean body weights and mean body weight gain for treated animals were comparable to non-treated animals. The results indicate Kv137-1036 is not pathogenic as clearance from the lungs, liver, cecum and lymph nodes was reported by day 15 of the 22-day observation period. Under the conditions of this study, the intra-tracheal administration of Kv137-1036 at a dose level containing 5.8 × 10* ^*8*^ *CFU showed no evidence of acute toxicity or pathogenicity.”*

The paper then states *“Results demonstrated complete clearance of Kv137-1036 cells from animal tissues. Taken altogether, the six biosafety studies indicate that Kv137-1036 does not pose acute toxicity or pathogenicity hazards to humans or animals following exposure” (p 3270*). Although this statement might be true, one pathogenicity study done in rats for 15 days via only one exposure pathway and dose could be seen as insufficient for release of the PROVEN product on millions of acres of a food crop.

A second article on safety frameworks is also authored by Pivot Bio personnel (Marian et al., 2024). It does not present new testing data, but rather reviews the existing literature on *K. variicola* pathogenicity and prevalence in infections, dismissing much of its importance in the context of agricultural exposure pathways. It concludes that

*“In the case of K. variicola strains developed for use as biofertilizers, the intended context is exclusively environmental, by way of application to crops. Revisiting the question of safety in light of this context leads to several observations. First, studies to date do not indicate infection risks in healthy humans, and virulence and antibiotic resistance factors, already found infrequently in K. variicola, would not encounter the acquisition pressures found in a hospital or community environment when applied in the field. Furthermore, these strains are not intended for direct human exposure, and application methods are designed to minimize exposure risk. Strains selected for field use were well characterized in terms of mechanism of action, genotype, and phenotype, and did not show toxicity or pathogenicity in a standard battery of in vivo biosafety tests.” (p. 06)* (Marian et al., 2024).

It should be noted that the battery of tests on pathogenicity to which the authors refer seems to be (according to public information) the one test described in Wen et al. (2021). Although Marian et al. (2024) make some valid points about potentially limited exposure routes (i.e., the product is applied to seed), humans could be exposed at application and harvesting, as well as through environmental pathways (e.g., water runoff into natural water bodies used for recreation). Also, antibiotic pressures exist in agricultural settings, as antibiotics are heavily used in livestock and appear in waterways (Wu et al., 2023). Thus, the potential for antimicrobial resistance genes to arise and be transmitted from and to *K. variicola* may be a real possibility. Furthermore, it seems warranted to test pathogenicity of PROVEN formulations 1) in different types of animals such as livestock or primates (i.e., only 32 rats were tested), 2) via more exposure routes other than inhalation (e.g., UTIs and wound infections occur from *K. variicola*), 3) in immunocompromised mammal models (Marian et al., 2024), and 4) in multiple replicates of studies looking at different endpoints (i.e., other than weight gain and organ colonization at one dose). *Klebsiella varricola* is an emerging pathogen of concern in medical literature and hospital settings with growing antibiotic resistance. Post-market monitoring of potential human, livestock, and wildlife pathogenicity due to *Kv137-1036* close to agricultural areas of application would also be prudent. In addition to no premarket federal, regulatory review, there is also no mandated federal surveillance program to track the long-term environmental persistence, mutations, or biogeochemical and health impacts of these GEMs.

Regardless, the company should be applauded for conducting safety studies, as they were not required by the federal regulatory system. That is the larger point of this article, in that the jurisdictional cracks in the CFRB and EPAs regulations under TSCA allowed the company to successfully scale a live, self-replicating human pathogen to millions of acres with very little (FDA voluntary consultation that cannot be seen) to no federal oversight.

As this case study illustrates, the definition of “intergeneric” is key to whether GEMs will be regulated for environmental release under TSCA, and perhaps regulated at all (if the GEM does not fall into other product categories like pesticides). In EPA’s 1997 regulations, which have not been officially modified since 1997, intergeneric organisms are defined as “organisms formed by combining genetic material from organisms in different genera” (EPA, 1997 CFR 40 C.F.R. §§ 725.1(a), 725.3). Wholly synthetic DNA gene sequences may not be considered intergeneric, as they do not come from a different taxonomical genus, but rather could be designed and constructed *de novo* in the lab and not found in nature. Scholars have pointed out this potential gap for GEMs containing synthetic DNA or xenobiology (Mandel and Marchant, 2014). EPA official regulations for microbial biotechnology have not been revised to clarify this issue since their promulgation in 1997 (EPA, 1997). However, in a more recent summary of the regulations put forth by EPA in 2012, the agency interprets the definition of intergeneric with regard to synthetic sequences in the following way:

*“The genetic sequence of the synthesized gene may be identical to a sequence known to occur in an organism in the same genus as the recipient microorganism. If so, the resulting microorganism is considered intrageneric and thus not new. Conversely, the sequence of the synthesized gene may be different or not known to be identical to a sequence in the genus of the recipient microorganism, in which case, the resulting product is considered intergeneric. EPA strongly encourages any manufacturer of a new microorganism using synthetic DNA to contact the Agency*.*”* (EPA, 2012).

This statement suggests EPA’s operational intent is to broadly interpret “intergeneric” to include wholly synthetic genes not found in nature as regulated under TSCA. Regardless, scholars have noted that the definition of intergeneric in the formal regulations is outdated, not accounting for whether regulatory or noncoding DNA sequences are included, frequent changes made in taxonomies for species and genera, and synthetic DNA and xenobiology (Mandel and Marchant, 2014; Chemla et al., 2025). Organisms could also be highly modified with several genetic edits, rearrangements, and modifications to endogenous or native genes with synthetic biology that could change the functions of the organism, and its risk profile, even if the changes are all intrageneric. For example, pathogenesis could be enhanced with intrageneric changes that are “not new” but impact microbial community dynamics. We return to recommendations for strengthening regulatory oversight in the discussion.

### GE tooth-protection bacteria

Lantern Bioworks has commercialized a GEM to prevent tooth decay. Tooth decay is caused by lactic acid producing bacteria. Native *Streptococcus mutans* is good at forming biofilms and producing lactic acid and thus is a significant contributor to dental cavities. To make the GEM, a strain of *Streptococcus mutans* BCS3-L1 was genetically engineered to replace the endogenous lactate dehydrogenase gene (making lactic acid) with an alcohol dehydrogenase gene (making alcohol) from *Zymomonas moblis* (Hillman et al., 2000; Hillman, 2002; Hillman et al., 2007). This transgenic modification was designed to prevent lactic acid production. The GEM produces alcohol instead of lactic acid and also secretes a compound called Mutacin-1140 which kills other strains of *Streptococcus mutans* but does not destroy BCS3-L1 (Hillman et al., 2000; Hillman, 2002; Hillman et al., 2007). The idea is that when the GEM is pasted onto teeth it will displace native *S. mutans* and lead to less lactic acid in the mouth and therefore less tooth decay (Hillman et al., 2000; Hillman, 2002; Szalinski, 2024; Chemla et al., 2025).

The commercialization pathway of the product, marketed as “Lumina Probiotic” (Lumina, 2025), is one of decades of development and testing which ended with regulatory avoidance by changing marketing claims. Initially, the company claimed that “a one-time brushing with this genetically modified bacterium could indefinitely prevent dental cavities” (Szalinski, 2024). Because of the original disease treatment claims of “preventing dental cavities,” the GEM initially fell under FDA drug/biologic jurisdiction and had to undergo clinical trials. The original company, Oragenics, filed a New Drug Application with the FDA and started clinical trials in 1998 (Pollack, 2004; Jarry, 2024; Szalinski, 2024). According to the lead scientist and original developer Dr. Hillman, in tests on rats, the GEM reduced cavities by approximately 50%, and in an early voluntary human trial, the antibiotic producing strain did replace wildtype *S. mutans* and persisted in their mouths for 15 years (Pollack, 2004).

However, in 2004, the FDA put additional constraints on the clinical trials due to concerns that the GEM could be transmitted between people, have unintended risks (e.g., from increased alcohol production in the mouth or changes to the oral microbiome), and revert back to the wild type strain causing more cavities ultimately (Pollack, 2004). Transmission between generations would also likely occur as children typically get *S. mutans* from their parents due to close contact during tooth development. One safety constraint FDA required involved modifying the GEM so an additional amino acid not typically found in food would be required for survival (Hillman et al., 2007). The human clinical-trial subjects would need to take the amino acid with the GEM paste, and then if transmission were to occur to non-clinical trial subjects, the GEM would theoretically die in the absence of that special amino acid. Another safety constraint placed by the FDA on the trials was that the human subjects should not have teeth, but dentures, so that they could be removed and cleaned if any unanticipated consequences arise (as permanent colonization of the mouth is the goal of the final product). Both of these constraints were intended to reverse the colonization and remove the GEM if any adverse consequences were to occur. For the FDA mandated trial, the company tried to recruit human subjects under the age of 55 with full dentures to participate and only found 1, whereas the FDA demanded over 100 (Jarry, 2024). They then proposed to the FDA a trial with participants who had actual teeth but were confined to a hospital setting (to prevent transmission outside of the trial), but they could not recruit participants for it (Jarry, 2024). This back and forth with the FDA lasted until 2014 before the company Oragenics and the developer Dr. Hillman gave up on the trials, and the patent for the GEM expired.

About 10 years later, in 2023, the company Lantern Bioworks made a deal with Oragenics to buy the rights to develop the product. Lantern Bioworks launched the product under the name Lumina Probiotic. Notably, in spring 2024, the company removed the claims about cavity prevention and oral disease treatment on its marketing materials and website. In an interview with a journalist, the founder of Lantern Bioworks, Aaron Sliverbrook, said that his lawyer advised changing the wording on the website so that the company could market the GEM as a cosmetic instead of a drug (Szalinski, 2024). New claims on the company’s current website state instead that “Lumina is a probiotic optimized through biodesign to rebalance your oral pH and inhibit the growth of acid-secreting bacteria, to protect enamel and freshen the mouth” (Lumina, 2025).

Under the FDCA, the FDA does not review cosmetics prior to marketing and only has recall authority should a problem arise during consumer use. The FDA does not pre-approve cosmetic products before they enter the public market, and they do not need to go through rigorous testing and trials as drugs do. The Modernization of Cosmetics Regulation Act of 2022 (MoCRA) did strengthen the post market authorities of FDA, however. Under MoCRA, FDA now has mandatory recall authority, and companies are required to list their product and its ingredients and track and report any serious adverse health events to the FDA. Regardless, with the removed cavity prevention claims, Lumina Probiotics, a GEM with significant enough health concerns to require rigorous FDA-approved clinical trials that were not completed, could enter the market freely with no premarket federal agency review.

The benefits of the Lumina Probiotic are also uncertain. Several bacteria cause tooth decay, not just *S. mutans*. For example, Philip et al. (2018) estimates that less than 2% of cavities are caused by the species and argue that an ecological approach to cavity prevention is warranted, one in which a health-promoting oral microbiome protects teeth from cavity-producing bacteria. Because Lumina Probiotic GEM also contains a gene for mutacin, an antibiotic which destroys bacteria, it is possible that application of the GEM to the teeth could ultimately increase cavities by degrading a protective oral microbiome. Because clinical trials evaluating effectiveness and safety have not been completed, these questions remain unanswered. Moreover, several potentially important risks have not been adequately studied, including effects on the health-protective oral microbiome from increased alcohol production by the GEM; effects on the gut microbiome from the antibiotic-producing GEM; the potential transfer of the antibiotic resistance gene to pathogenic bacteria in the mouth, gut, or environment; and the long-term consequences of repeated, small increases in blood alcohol concentration resulting from the GEM. Although the company dismisses or minimizes these concerns (Alexander, 2023), they have not been rigorously evaluated. In addition, informed consent issues or risks also arise from the transmission of the bacteria to those not undergoing treatment through kissing or sharing utensils (Alexander, 2023; Jarry, 2024; Szalinski, 2024). The product is designed to permanently colonize the mouth, so irreversibility if any adverse effects appear is an issue.

Regardless, the product has been applied to humans who were early volunteers outside of FDA-approved trials and on an island in Central America where regulations were not required (Pollack, 2004; Jarry, 2024). It is unknown whether the product is currently widely sold, as the link to pre-order the product does not seem to be active although the website is active (https://www.luminaprobiotic.com/). The larger point is that this GEM represents another regulatory void, one where changing the claims on a product can evade pre-market safety trials and federal regulation, even when potential risks are present.

This case study highlights a major problem for future applications of advanced synthetic biology approaches to GEMs. Currently, xenobiology, synthetic DNA sequences, and highly synthetic organisms would not be regulated at all if the products were considered as cosmetics. As the National Academies points out in its recent study, synthetic cells “that incorporate non-traditional biochemistries, alternative genetic codes, or hybrid biological-chemical architectures may challenge existing detection and monitoring methods, empirical benchmarks, and modeling assumptions” (NASEM, 2026, p 5). Assessing risk of such consumer products would not be required prior to market release, and these challenges in detection and monitoring would make post market monitoring for any adverse events more difficult. FDA regulation would be limited to post-market recall, and health effects of highly synthetic GEMS may go undetected until health damage occurs.

### GE hangover-prevention bacteria

In 2019, a living GEM was marketed to treat hangovers. However again, as in the case study above, the company’s official claims allowed them to avoid characterizing the product as treating disease or improving health, thereby bypassing premarket regulatory reviews and oversight. ZBiotics developed and commercialized an engineered *strain (ZB183)* that would be consumed orally and deliver active enzymes to the gut to breakdown acetaldehyde, a toxic byproduct of alcohol metabolism and a main offender of hangover symptoms (Chemla et al., 2025; Snitkoff and David, 2026). ZB183 constitutively expresses an acetaldehyde dehydrogenase enzyme from *Cupriavidus necator* (Appala Naidu et al., 2019). It does not appear that common transgenes used in GEMs production, antibiotic resistance genes, are present in ZB183 (Hassan-Casarez et al., 2024), as the published safety study (Appala Naidu et al., 2019) does not state that any other transgenes are present. Over 15 million shots of the liquid product have been sold as of 1 July 2026, and the product is available for purchase over Amazon (Watson, 2026).

The regulatory line was crossed when the marketing approach of treating or preventing a hangover (as a disease or medical condition) were changed in favor of a functional or probiotic food. The company did not have to go through the rigorous FDA pre-market framework for drugs or biologics under the FDCA (i.e., regulated clinical trials), but rather went through the FDA food regulatory framework under which has a couple of different regulatory pathways. One regulatory pathway for new food ingredients (NFI) is to consider them as food additives (as discussed above). Food additives undergo premarket testing for safety under FDA surveillance through a petition process. Data is required on chemical composition, manufacturing, purification, and toxicological and safety information. After FDA provides input on this information, the food additive petition is open for public comment in the Federal Register, and FDA may also put restrictions or limits on the additive’s labeling and use. However, under Section 201(s) of the FDCA, a food additive can escape this extensive, pre-market FDA evaluation if it is “generally recognized” as safe by qualified experts. There are two pathways for NFI GRAS categorization—voluntary GRAS notification to FDA by the company or a complete self-determination of GRAS status by the company itself with no FDA notification (Chemla et al., 2025; Brewer et al., 2026; Snitkoff and David, 2026). ZBiotics appears to have used the second, more lenient GRAS self-affirmation process for ZB183 as the product does not appear on FDA’s GRAS notification database (Snitkoff and David, 2026). This GRAS pathway does not require notice to or input from the FDA in contrast to the voluntary GRAS notification to FDA pathway.

GRAS determinations assume that the substance is determined safe by qualified experts or shown to be safe through scientific testing. The company did convene a board of experts (composition unknown to authors) to review the one *in vivo* toxicology study conducted by company personnel, which is published in the peer-reviewed literature (Appala Naidu et al., 2019). It appears as if *in vitro* toxicity and antibiotic-resistance gene transfer studies were also conducted by the company, but those results do not appear to be published in the literature other than on the company website (Zbiotics, 2026). It is commendable that the company chose to publish the *in vivo* toxicity study, as under the self-affirmed GRAS regulatory option that the company took, it is not required to generate safety data.

The *in vivo* safety study described in Appala Naidu et al. (2019) was a 90-day toxicity test done on rats with 1x, 10x, and 100x expected commercial dose levels for 90 consecutive days. The results are summarized as follows:

“*B. subtilis* ZB183 had no effects on clinical signs, mortality, ophthalmological examinations, functional observational battery, body weights, body weight gains and food consumption in both sexes. There were no test item-related changes observed in haematology, coagulation, urinalysis, thyroid hormonal analysis, terminal fasting body weights, organ weights, gross pathology and histopathology. A minimal increase in the plasma albumin level was observed at 10^10^ and 10^11^ CFU/kg/day doses without an increase in total protein in males or females and was considered a nonadverse effect.” (p. 1) (Appalla-Naidu et al., 2019).

Although these studies point to safety to rats for these endpoints, human trials may have uncovered other adverse or side effects important to consumers that they could articulate to the developers. Had the probiotic undergone the drug approval route, human clinical trials would have been required by the FDA. The majority of purchasers of the product on Amazon.com give favorable reviews; however, there are others who report nausea, bloating and cramps, as well as the product not working well or increased hangover symptoms (Amazon, 2026). Active and structured post-marketing monitoring of these side effects is not required by federal regulatory agencies, and the authors are uncertain if the company is doing so passively or actively. There is also the chance that ZB183 could reach the environment through waste streams and could be transmitted to other people without their consent, as these risks have not been studied (Snitkoff and David, 2026).

The risk of anti-microbial resistance (AMR) gene transfer to ZB183 was also considered by Zbiotics (2026). Hassan-Casarez (2024) describe the construction of the engineered strain and that it was “confirmed to be free of virulence factors, toxins associated with pathogenicity, and of transferable antibiotic resistance genes, consistent with the known traits of *B*. *subtilis* PY79”. This study, in addition to the toxicity study, was also coauthored by Zbiotic personnel. ZBiotics (2026) states on the company’s website that they tested ZB183 *“for (extrinsic/acquired resistance) by genome sequencing and by phenotypic testing (i.e., testing their growth in the presence of antibiotics) and none were found”.* However, the methods of that test and whether they mimic a human gut setting are not detailed in Hassan-Casarez (2024) or on the website (Zbiotics, 2026). The transfer of AMR genes between microbes in the human gut is pervasive based on the peer-reviewed literature (e.g., McInnis et al., 2020). Whether AMR genes could be transferred between human pathogens and Z183, and then *vice versa*, in a human gut setting is unknown, although the strain would not appear to be the origin of such genes based on the company’s unpublished tests.

In summary, the company did one *in vivo* toxicity safety test in rats, convened an expert panel to review the rat study, and proudly labels the product as GMO and genetically engineered. The company also states a commitment to safe genetic engineering and engages with regulatory and academic audiences at conferences (Zbiotics, 2024). All these efforts can be applauded, as they are not mandated by federal regulation in the GRAS NFI pathway. However, by intentionally framing the GEM as probiotic food ingredient, rather than a cure for a medical condition (hangover), ZBiotics was able to escape human trials that would have been required under the FDA drug/biologics approval pathway and could have better determined whether the product is effective and safe in the human digestive tract and environment. This marketing and regulatory approach seems to limit their commitment to responsible and safe development. Without their publication, transparency of the *in vitro* safety tests is lacking, and so is information about the composition and deliberations of the expert panel. Greater transparency in these areas would support a commitment to responsible development.

This case study highlights another fundamental regulatory gap for the future of synthetic organisms. A company producing a food-based GEM with wholly synthetic DNA sequences or xenobiology, or one highly engineered with synthetic biology (whether intra- or intergeneric) could self-determine the product’s GRAS status with no FDA notification. In the case of Zbiotics, the GEM was not highly engineered and the components were well-known, but this would not be the case with synthetic GEMs. The question remains whether mandatory FDA notification of GRAS or food additive petitions should be mandated for all GEMs due to the increasing novelty and complexity that synthetic biology enables. During the writing of this manuscript, on 11 August 2026, FDA proposed new GRAS regulations that would require mandatory notification to the agency for GRAS determinations (FDA-DHHS, 2026). This proposed rule is under the public comment period and is discussed in more detail below.

## Discussion

The three case studies highlight important gaps in the regulatory system which are summarized in Table 1.

**TABLE 1:** Regulatory and safety features of the GEM case studies.

Microbial and regulatory profile	Pivot bio (PROVEN® 40)	Lantern bioworks (lumina)	ZBiotics (pre-alcohol)
Microbial strain	*Kosakonia sacchari*/ *Klebsiella variicola*	*Streptococcus mutans* (BCS3-L1)	*Bacillus subtilis* (ZB183™)
Function	Excretes ammonia to replace synthetic fertilizer	Converts sugar to alcohol to prevent lactic acid decay	Expresses enzyme to break down toxic acetaldehyde
Primary regulatory agency	EPA (office of pollution prevention and toxics) No review “intrageneric”	FDA (office of cosmetics and colors) No pre-market review	FDA (office of food chemical safety, dietary supplements, and innovation) No premarket review
Final classification	Crop nutrition/ Non-pest soil inoculant	Cosmetic oral probiotic	New food ingredient (GRAS)
Pre-market testing	None Exempt from federal transgenic GMO rules—states require registration however	None Avoided drug approval path by altering claims	None Avoided drug approval with limited claims and avoided food additive petition with GRAS self-determination and no FDA notification

### Strengthening regulatory oversight without new legislation

Each of the above cases of living GEMs in environmental or consumer products arguably presents safety issues that would warrant further scrutiny by the federal agencies. PROVEN is formulated with an emerging human pathogen and spread widely in the environment. Lumina could change dental microbiome composition, increase alcohol production in the mouth, and be transferred among people. ZBiotics may change gastrointestinal flora and also be transmitted through the environment. None of these have undergone pre-market federal regulatory review.

While many GEMs are well-covered by the U.S. CFRB, such as pesticides, drugs, plant pests, and food additives, and arguably even over covered (e.g., plant pests used as pesticides like CTV are regulated by USDA under PPA and EPA under FIFRA, both which require premarket risk or safety assessments) (NSCEB, 2026), others evaded oversight of an outdated system based on old, product-based and product-claims statutes (NSCEB, 2026). The statutes were written for chemicals in most cases (other than the Plant Protection Act), not living modified organisms. There have been recent national calls for updates to the CFRB (NSCEB, 2026), and for updates to modernize GEMs regulation (e.g., FOE, 2023; LCSSP, 2024; Shams et al., 2024; Snitkoff and David, 2026; Brewer et al., 2026). Although there have been some changes to the CFRB over time, these efforts have arguably been missed opportunities for true structural reform or for the passage of new national biotechnology laws and associated regulations (Kuzma, 2016). With the lack of political will or Congressional attention to pass new biotechnology law(s) with clear reform intentions continuing, some recommendations for plugging the current gaps for GEMs are summarized below.

First, claims of a product that works for an obvious purpose (e.g., for improving health or treating disease) should not be able to be altered by companies (e.g., for a cosmetic purpose) in order to avoid regulation (e.g., Lumina case). The agencies should be more aggressive in monitoring and legally challenging these claims. Second, food ingredients with living GEMs should default to a food additive petition or FDA-mediated GRAS notification given their persistence, survival, growth and transmission in the environment and within and between humans (e.g., ZBiotics case). FDA could amend its GRAS regulations accordingly without new legislation required (Snitkoff and David, 2026), and the agency is currently attempting to do so. There is a proposed new GRAS rule, currently under public comment period, that would require FDA notification for GRAS determinations, essentially doing away with the self-determination GRAS pathway that was used in the Zbiotics case (FDA-DHHS, 2026). This seems to be a step in the right direction for emerging GEMs in foods and would allow for FDA to provide a more independent view on the adequacy of safety studies done for GRAS determinations (i.e., in comparison’s to the company’s self-review of adequacy). The proposed rule, if enacted, would also expand the existing public inventory of submitted GRAS notifications, improving consumer transparency and increasing FDA’s ability to track ingredients entering the food supply in case post-market oversight is required (FDA-DHHS, 2026).

Third, a unified regulatory submission portal for all biotech products that sits above individual federal agencies should be used to ensure coordination between agencies, prevent overregulation, and avoid under regulation resulting in no pre-market testing or agency review (e.g., NASEM, 2017; Shams et al., 2024). This would likely require only agency administrative action. Fourth, the EPA should revise its regulations under TSCA for environmental release of GEMs to include “intrageneric” GEMs (e.g., the PROVEN case study). Because GEMs are regulated as “toxic chemicals” under the CFRB, and scientific evidence indicates that the source of the gene for genetic engineering is not correlated with toxicity or risk (e.g., NRC, 2000; NASEM, 2016), limiting regulation to intergeneric GEMS lacks a clear scientific basis and appears arbitrary. NASEM (2026) addressed this challenge for synthetic cells by using a continuum-based framework that evaluates systems according to structural and functional characteristics, including genetic content and replication capacity, together with contextual factors such as intended use, deployment environment, persistence, and interactions with living systems. Applied to GEMs, this approach suggests that risk-relevant biological characteristics and exposure contexts may provide a more durable basis for oversight than categorical distinctions based primarily on taxonomic origin or the source of engineered genetic material.

These are just a few of the formal oversight changes that seem warranted considering the above-mentioned cases. Lastly, transparency and independence of safety studies could be promoted and perhaps incentivized by federal agencies as a policy change (e.g., all case studies). The cases highlight a lack of transparency (e.g., composition of expert panels, lack of fully published or described safety studies, etc.) and independence (e.g., safety studies co-authored, co-authored, and/or overseen by the company producing the GEMs).

Arguably, the federal agencies could implement the above without new federal legislation—either through regulatory changes, administrative actions, or policy guidance. The CFRB is flexible and adaptive (e.g., see Kuzma et al., 2009), having increased or decreased coverage for certain GEOs and GE products over its 40-year implementation without any new laws (Kuzma, 2023). For example, GE animals were decided to be regulated as New Animal Drugs without new statutes or legislation, and just through FDA policy guidance; GE insects have been divided between the FDA (as drugs) and EPA (as pesticides) depending on purpose without new statutes or regulations (FDA/EPA MOU); USDA has proposed regulatory revisions for GE plants that included Plant Protection Act weediness provisions (2016) or excluded them (2020) depending on the political administration; and USDA decided to exempt all GE plants without plant pest sequences through an “Am I regulated letter of inquiry” process when gene editing came to be used to generate them (Kuzma, 2023) even though in early days of biotechnology the agency said it would capture all GE plants under the PPA through a broad interpretation of the definition of “plant pest” (NRC, 2000).

### Broader risk-proportionate recommendations for improving oversight of living GEMs

The case studies suggest that the central regulatory problem is that the Coordinated Framework can impose substantially different requirements on products with potentially comparable risk characteristics because jurisdiction depends on legacy statutory categories, the source of genetic material, and the claims used to market a product. At the same time, some well-characterized and contained GEMs may be subject to requirements that are disproportionate to their plausible risks. Reform should therefore seek to close consequential gaps while reducing duplicative or unnecessary review. The guiding principle should be that oversight is proportionate to the biological characteristics of the organism, the context and scale of its use, and the nature of potential exposure, rather than determined primarily by whether a modification is intergeneric or intrageneric or by the commercial category selected for the product (National Academies of Sciences, Engineering, and Medicine [NASEM] 2016; 2017; National Research Council [NRC] 2000; National Security Commission on Emerging Biotechnology [NSCEB] 2026).

The characteristics of living GEMs require particular attention. Unlike conventional chemical products, microorganisms may survive, reproduce, evolve, exchange genetic material, interact with microbial communities, and move beyond their intended place of use. These characteristics do not establish that a product is unsafe, but they affect the kinds of evidence needed to evaluate safety and the adequacy of regulatory categories developed primarily for static products. Premarket review should therefore be triggered by plausible pathways to harm, including persistence, replication, transmission, horizontal gene transfer, pathogenicity, ecological disruption, effects on host-associated microbiomes, and the difficulty of reversing an intervention after release, as well as an evaluation of potential benefits (such as efficacy) against risks (Brewer et al., 2026; Chemla et al., 2025; Linde Center for Science, Society, and Policy [LCSSP] 2024; NSCEB, 2026; Shams et al., 2024). It should be noted, however, that there are still sufficient challenges to obtain empirical data on environmental fate, persistence, detection, and gene transfer of GEMs, and hence there is a need to develop the empirical tools and infrastructure to measure and detect GEMs in various environments and products to character potential risks (NASEM, 2026).

Table 2 presents a set of reforms derived from the regulatory gaps identified across the three case studies. The recommendations are organized into six categories: regulatory scope, interagency coordination, premarket evidence, transparency and independence, post-market oversight, and claims and informed choice. Together, these reforms would create a minimum governance structure in which commercially released GEMs are identified, directed to an appropriate regulatory pathway, evaluated using risk-proportionate evidence, and monitored when meaningful uncertainty remains. The NASEM (2026) report on synthetic cells recommended a somewhat similar approach, however, for living GEMs, this type of coordination could support the proposed federal notification system by helping agencies determine whether a product warrants exemption, streamlined review, or more extensive evaluation. This could improve coordination across existing authorities and reduce the likelihood that novel products receive little or no review because they fall between regulatory jurisdictions.

**TABLE 2:** Recommended reforms for federal oversight of living genetically engineered microorganisms.

Reform category	Recommended action	Regulatory rationale
Risk-based regulatory scope	Require federal notification before the commercial use or intentional environmental release of a living GEM. Replace EPA’s intergeneric trigger with a tiered framework based on the organism, engineered trait, intended use, exposure, persistence, and scale of release	Notification would allow agencies to identify products that fall outside existing categories. Tiered review would focus oversight on plausible risks while supporting expedited review of well-characterized, lower-risk applications
Interagency coordination	Create a unified federal entry point that determines jurisdiction, identifies the lead agency, coordinates additional review, and documents exemptions	A common entry point would reduce duplicative oversight and prevent products from receiving no review because they fall between agency authorities
Premarket evidence	Establish tiered data requirements addressing pathogenicity, horizontal gene transfer, antimicrobial resistance, ecological effects, persistence, replication, transmission, reversibility, and effects on host-associated microbiomes	Evidence requirements should reflect the scale and nature of plausible exposure. Products intended for widespread release, persistent colonization, or use in sensitive environments should receive greater scrutiny
Product classification	Require notification and risk-proportionate review for food ingredients containing living GEMs rather than relying solely on undisclosed self-determined GRAS conclusions. Consider foreseeable function and biological activity alongside express marketing claims when assigning a regulatory pathway	Living GEMs may persist, replicate, interact with the human microbiome, enter waste streams, or reach nonusers. Removing a disease-treatment claim should not eliminate review when the organism’s function and exposure remain substantially unchanged
Transparency and independent evaluation	Create a public registry of commercial and environmentally released GEMs. Disclose the organism, modification, intended use, regulatory status, basis for exemptions, available safety evidence, study funding, authorship, and relevant conflicts of interest	Publicly available information would allow independent assessment of product classification, supporting evidence, and safety conclusions
Post-market oversight	Require proportionate monitoring and periodic reassessment when a GEM is released at scale, is intended to persist or colonize, may reach nonusers, or presents unresolved uncertainty. Permit agencies to modify conditions of use when evidence or exposure changes materially	Continuing oversight would help identify delayed or emerging effects involving persistence, microbial-community change, gene transfer, antimicrobial resistance, unintended transmission, or adverse health outcomes
Labeling and informed choice	Require consumer products to disclose clearly that they contain a living GEM and, when relevant, provide information about persistence, transmission, reversibility, and material uncertainties	Clear disclosure would support informed choice, particularly when use may expose people who did not directly select the product

These recommendations distinguish baseline notification from full premarket review. Notification would allow agencies to determine whether a product qualifies for an exemption, streamlined review, or more extensive evaluation. These recommendations should be implemented through a combination of administrative and legislative action. Under existing authorities, federal agencies may be able to improve interagency referral procedures, publish clearer guidance, standardize data expectations, increase the transparency of regulatory determinations, and coordinate review across overlapping programs. As discussed above, the history of the Coordinated Framework demonstrates that agencies have repeatedly interpreted and adjusted existing authorities in response to new biotechnology products (Kuzma et al., 2009; Kuzma, 2023). However, agencies should not be presumed to possess sufficient authority to resolve every gap identified from the cases or implement all the changes identified in Table 2. For example, requirements that materially expand pre-market authorities to consumer or dietary supplement products (without health or disease treatment purposes) or to establish new post-market surveillance responsibilities may require congressional authorization.

A tiered approach would help avoid replacing regulatory gaps with indiscriminate regulatory expansion. A baseline notification system should not subject every GEM to the requirements used for a new drug, pesticide, or high-risk environmental release. Well-characterized organisms with a documented history of safe use, limited persistence, effective containment, and low exposure could qualify for exemptions or expedited review. More extensive evidence and continuing oversight would be appropriate when a GEM uses a pathogenic or poorly characterized organism, is intended to reproduce or persist, is released across large geographic areas, permanently colonizes a human host, may be transmitted to non-users, or cannot be readily recalled or reversed. This approach would direct regulatory resources toward novel or consequential risks while providing clearer pathways for lower-risk products (NASEM, 2017; NSCEB, 2026).

Scale of deployment should also influence the level of oversight. A microbial product used in a limited setting presents a different exposure profile from the same organism deployed across millions of acres or intended to persist within a human microbiome. Regulatory requirements should therefore be capable of increasing as deployment expands, particularly when wider use increases opportunities for persistence, transmission, ecological interaction, or exposure of non-users.

The recommendations are intended as minimum components of a more coherent federal approach rather than a complete redesign of biotechnology governance. Their purpose is to ensure that living GEMs do not receive no federal review solely because of their taxonomic construction, their placement within a legacy product category, or narrowly framed commercial claims. At a minimum, the federal system should be able to identify which living GEMs have entered commerce, determine which agency is responsible, evaluate them using evidence proportionate to plausible risks, disclose the basis for regulatory decisions, and monitor products when persistence, transmission, scale, or uncertainty warrants continuing oversight.

The need for clearer and more adaptive oversight extends beyond the three commercial products examined here. Regulatory pathways remain uncertain for emerging GEM applications in the built environment, including living construction materials, indoor probiotics, and engineered microorganisms designed to detect or suppress human pathogens in hospitals, homes, and other shared spaces (Hardwick et al., 2024; Landreville et al., 2026). Survey research with U.S. adults indicates that inadequate regulation is a prominent concern surrounding the use of microbiome engineering in built environments (Cummings et al., 2024). Trust in the regulatory system and in the institutions responsible for developing and managing these technologies are also important predictors of public acceptance across proposed indoor applications (Cummings C. L. et al., 2025; Cummings et al., 2025 C.; 2026). Accordingly, responsible governance will require more than technical risk assessment. Transparency, independent evaluation of safety evidence, public and stakeholder engagement, and attention to informed consent, ownership, and privacy will also be important for responsible development and public trust (Landreville et al., 2026).

Having a more coordinated oversight and governance approach for GEMs has also been recommended in a recent NASEM report on synthetic cells, which calls for a unified governance strategy led by the White House Office of Science and Technology Policy (OSTP) and in coordination with the National Security Council (NSC) (NASEM, 2026). In addition to a harmonized national strategy, international coordination and data-sharing is also important to support public confidence in oversight (NASEM, 2026). As GEM applications move from controlled settings into broader consumer, environmental, and built-environment uses, oversight mechanisms will also need to adapt as evidence, exposure pathways, and the scale of deployment change. This would allow regulatory expectations to remain proportionate to both technological development and real-world use. Overall, clear and credible governance will be necessary to support public confidence in GEMs used in consumer products, environmental applications, and shared built environments, especially as GEMs become more synthetic and highly engineered as a result of advances in xenobiology and synthetic biology (NASEM, 2026).

## Footnotes

1 In this article, genetic engineering or GE refers to both transgenic modification with the introduction of genes from other species and the modification of endogenous genes using modern biotechnology methods such as CRISPR gene-editing. Genetic engineering encompasses the use of modern biotechnology approaches to alter or introduce genes. This definition is consistent with national and international regulatory definitions of the use of the term. For example, the National Human Genome Research Institute definition is “Genetic engineering (also called genetic modification) is a process that uses laboratory-based technologies to alter the DNA makeup of an organism. This may involve changing a single base pair (A-T or C-G), deleting a region of DNA or adding a new segment of DNA”.
